# Optimised photodynamic diagnosis for transurethral resection of the bladder (TURB) in German clinical practice: results of the noninterventional study OPTIC III

**DOI:** 10.1007/s00345-016-1925-0

**Published:** 2016-08-30

**Authors:** Thorsten Bach, Patrick J. Bastian, Andreas Blana, Angelika Kaminsky, Stefan Keller, Thomas Knoll, Christoph Lang, Soeren Promnitz, Burkhard Ubrig, Thomas Keller, Bryan Qvick, Maximilian Burger, for the OPTIC III Study Group, Akhmat Shabayev, Alexander Bick, Amir Hamza, Andreas Blana, Armin Secker, Benjamin Lorch, Bernd Hoschke, Christian Gilfrich, Christian Wülfing, Christoph Lang, Christoph Wiesner, Dirk Fahlenkamp, Dirk Scheer, Gero Diefenbach, Hans W. Wechsel, Herbert Leyh, Herbert Sperling, Lukas Manka, Marc-Oliver Grimm, Markus Funk, Sebastian Wille, Markus Wöhr, Michael Rink, Raik Wustmann, Roberto Olianas, Roland Steiner, Roman Flühr, Stefan Machtens, Stefan Vögele, Theodor Klotz

**Affiliations:** 1 Urologisches Zentrum Hamburg, Asklepios Kliniken Hamburg, Hamburg, Germany; 2 Klinik für Urologie, Marien Hospital, Düsseldorf, Germany; 3 Klinik für Urologie und Kinderurologie, Klinikum Fürth, Fürth, Germany; 4 Klinik für Urologie, Kliniken Maria Hilf GmbH Mönchengladbach, Mönchengladbach, Germany; 5 Klinikum Garmisch-Partenkirchen GmbH, Urologie, Garmisch-Partenkirchen, Germany; 6 Urologische Klinik Sindelfingen, Sindelfingen, Germany; 7 Klinik für Urologie, Knappschaftskrankenhaus Sulzbach, Sulzbach, Germany; 8 Klinik für Urologie, Klinikum Frankfurt (Oder), Frankfurt, Germany; 9 Klinik für Urologie, Augusta-Krankenanstalt GmbH, Bochum, Germany; 10 ACOMED Statistik Leipzig/Mediveritas GmbH München, Munich, Germany; 11 Ipsen Pharma GmbH, Ettlingen, Germany; 12 Klinik für Urologie, Universitätsklinikum Regensburg, 93053 Regensburg, Germany

**Keywords:** Blue light cystoscopy, Hexaminolevulinate, Non-muscle invasive bladder cancer, Observational studies, White light cystoscopy

## Abstract

**Purpose:**

White light cystoscopy (WLC) is the standard procedure for visualising non-muscle invasive bladder cancer (NMIBC). However, WLC can fail to detect all cancerous lesions, and outcomes with transurethral resection of the bladder differ between institutions, controlled trials, and possibly between trials and routine application. This noninterventional study assessed the benefit of hexaminolevulinate blue light cystoscopy (HALC; Hexvix^®^, Ipsen Pharma GmbH, Germany) plus WLC versus WLC alone in routine use.

**Methods:**

From May 2013 to April 2014, 403 patients with suspected NMIBC were screened from 30 German centres to perform an unprecedented detailed assessment of the additional detection of cancer lesions with HALC versus WLC alone.

**Results:**

Among the histological results for 929 biopsy samples, 94.3 % were obtained from suspected cancerous lesions under either WLC or HALC: 59.5 % were carcinoma tissue and 40.5 % were non-cancerous tissue. Of all cancer lesions, 62.2 % were staged as Ta, 20.1 % as T1, 9.3 % as T2, 7.3 % as carcinoma in situ (CIS), and 1.2 % were unknown. Additional cancer lesions (+6.8 %) and CIS lesions (+25 %, *p* < 0.0001) were detected by HALC plus WLC versus WLC alone. In 10.0 % of patients, ≥1 additional positive lesion was detected with HALC, and 2.2 % of NMIBC patients would have been missed with WLC alone. No adverse events were observed.

**Conclusions:**

The results of this study demonstrate that HALC significantly improves the detection of NMIBC versus WLC alone in routine clinical practice in Germany. While this benefit is statistically significant across all types of NMIBC, it seems most relevant in CIS.

## Introduction

Suspected non-muscle invasive bladder cancer (NMIBC) is diagnosed using white light cystoscopy (WLC), followed by biopsy [1]; however, WLC can fail to detect 4–41 % of papillary Ta and T1 tumours, carcinoma in situ (CIS), dysplasia, multifocal growth, and microscopic lesions [2, 3]. Visualisation of bladder lesions including residual tumour tissue, small tumours, and CIS during transurethral resection of the bladder (TURB) can be improved with hexaminolevulinate blue light cystoscopy (HALC; Hexvix^®^, Ipsen Pharma GmbH, Ettlingen, Germany) [3–6]. Results from randomised controlled trials (RCTs) show that WLC plus HALC can detect an additional 7–30 % of cancer lesions versus WLC alone [6–11].

The quality of TURB and outcomes achieved in clinical studies can vary considerably [6]. Therefore, observational studies reflecting routine clinical practice are useful to assess the benefit of HALC outside of RCTs, since the effect of photodynamic diagnosis may differ between these settings. Prior observational studies conducted in Italy [7], Spain [8] and France [12] showed that in daily clinical practice, HALC can enhance the diagnostic accuracy of WLC, resulting in a lower tumour recurrence rate. Detection rates again varied, potentially reflecting different clinical settings between series.

Because of the observed differences in different countries, and as changes in EU regulations promote post-authorisation studies in different countries in Europe [13], we conducted a study to assess additional detection of cancerous lesions with HALC plus WLC versus WLC alone in patients with NMIBC undergoing TURB in routine clinical practice in various urology departments in Germany in unprecedented detail.

## Patients and methods

### Study design

During this multicentre, prospective, noninterventional study, patients with suspected NMIBC undergoing TURB in daily clinical practice were screened from 30 inpatient surgical urological German centres between May 2013 and April 2014. Centres utilising HALC were prospectively selected for inclusion. All enrolled patients received HALC and WLC.

The study followed recommendations of the International Epidemiological Association Guidelines for the Proper Conduct in Epidemiologic Research, and the International Society for Pharmacoepidemiology, Good Pharmacoepidemiological Practice Guidelines. Applicable local independent ethics committee and institutional review board approvals were obtained before study initiation.

The primary objective was to assess additional detection of NMIBC with HALC compared with WLC alone based on lesions in patients undergoing TURB, by analysing the detection rate with HALC and WLC versus WLC alone.

### Patients

All patients with suspected NMIBC and indication for TURB were included. Exclusion criteria included: repeat TURB (control TURB) 4–6 weeks after initial TURB, and Bacillus Calmette–Guérin (BCG) or mitomycin/intravesical chemotherapy instillation therapy ≤12 weeks before TURB. Inclusion and exclusion criteria followed recommendations in the HALC Summary of Product Characteristics (SmPC) [14]: contraindications include porphyria and hypersensitivity to the active substances/any of the excipients [14].

### Procedure

HALC and WLC were performed during the same surgical procedure after administration of hexaminolevulinate, according to the SmPC and local clinical practice procedure.

Hexaminolevulinate solution (50 ml of 8 mmol/l) should be instilled into the bladder via a catheter and retained for ≥1 h. After emptying the bladder, examination using blue light should be started <3 h after instillation. The entire bladder was examined and mapped using white light, then blue light, and biopsies of mapped lesions were taken [14, 15].

### Lesion detection

In each patient, lesions detected with WLC were numbered and their location recorded in a detailed bladder map; this was repeated using HALC. It was noted if lesions were detected with HALC and/or WLC, and whether lesions were confluent or not. Directly following cystoscopy, findings were recorded in a detailed online system, thereby minimising delay between the procedure and documentation.

### Histology

Histology was performed by the local pathology team of the participating centre, and results were added to the online patient records at a later stage. Lesions were resected using TURB, and biopsies were taken for histological evaluation by local pathologists. The biopsy method—cold biopsy or resection—was recorded. Lesions were histologically classified as carcinoma, dysplasia, hyperplasia, healthy tissue, inflammation, or unknown.

Carcinoma lesions were staged according to the tumour, nodes, metastasis classification of malignant tumours, and graded using the WHO classification (1973 and 2004). Individuals with confirmed tumour stage Ta, T1, or CIS were assigned to the European Organisation for Research and Treatment of Cancer (EORTC) risk scores (i.e. estimated probability of recurrence and progression in patients with NMIBC).

### Adverse event monitoring

All related adverse events (AEs; serious and non-serious) were reported as spontaneous cases.

### Monitoring

For each patient, an electronic case report form (eCRF) was used; into this eCRF, the investigator entered the collected data, the validity of which they were responsible for. Upon entry, data were automatically checked for completeness and plausibility. Random monitoring to confirm the study was being conducted in compliance with the protocol, and to verify data were being accurately reported on the eCRF, was conducted in approximately 10 % of the participating centres.

### Statistical analysis

Efficacy analysis was at a patient level and lesion level in the per protocol (PP) population, which included all treated patients in the intent-to-treat (ITT) population with no major protocol deviation, and valid cystoscopy and pathology/histology results. The safety analysis was performed on the ITT population (i.e. all enrolled patients).

As the study was exploratory, lesions within patients were considered quasi-independent (no clustered analysis).

The ratio of true-positive and false-positive fractions (rTPF and rFPF) was calculated from measures of sensitivity (=TPF) and specificity (=1-FPF) for HALC and WLC, respectively. The TPF was the proportion of lesions or patients positively tested during cystoscopy out of all lesions or patients, respectively, with histologically confirmed carcinoma. The FPF was the proportion of lesions or patients positively tested during cystoscopy out of all lesions or patients, respectively, with histologically confirmed non-carcinoma tissue.

Detection rate was defined as the proportion of lesions or patients with lesions detected during the procedure out of all lesions or patients, respectively. By definition, detection rate was 100 % for HALC plus WLC.

The primary efficacy endpoints were rTPF and rFPF (with 95 % confidence intervals [CI]) for HALC plus WLC versus WLC alone. The McNemar test was used to test the null hypothesis of equal sensitivities (rTPF = 1). rTPF was estimated (with 95 % CI) to assess the primary objective by evaluating the impact of HALC plus WLC versus WLC alone on the detection of NMIBC. Secondary efficacy endpoints were to assess detection rates with HALC plus WLC versus WLC alone for risk groups according to EORTC scores. Detection and false-positive rates were also compared on a patient level between HALC plus WLC and WLC alone. Pearson–Clopper 95 % CIs were calculated for these secondary efficacy endpoints.

The planned sample size was 364 patients using the assumptions: discordant test results between HALC plus WLC versus WLC alone of 12, 80 % power, 5 % alpha level, a fraction of the smallest subgroup investigated (15 %), and patients with missing histology (13 %).

Post hoc analysis included assessment of additional CIS lesion detection with HALC plus WLC versus WLC alone and of the number needed to treat for one patient to benefit from additional examination with HALC.

## Results

**Fig. 1:**
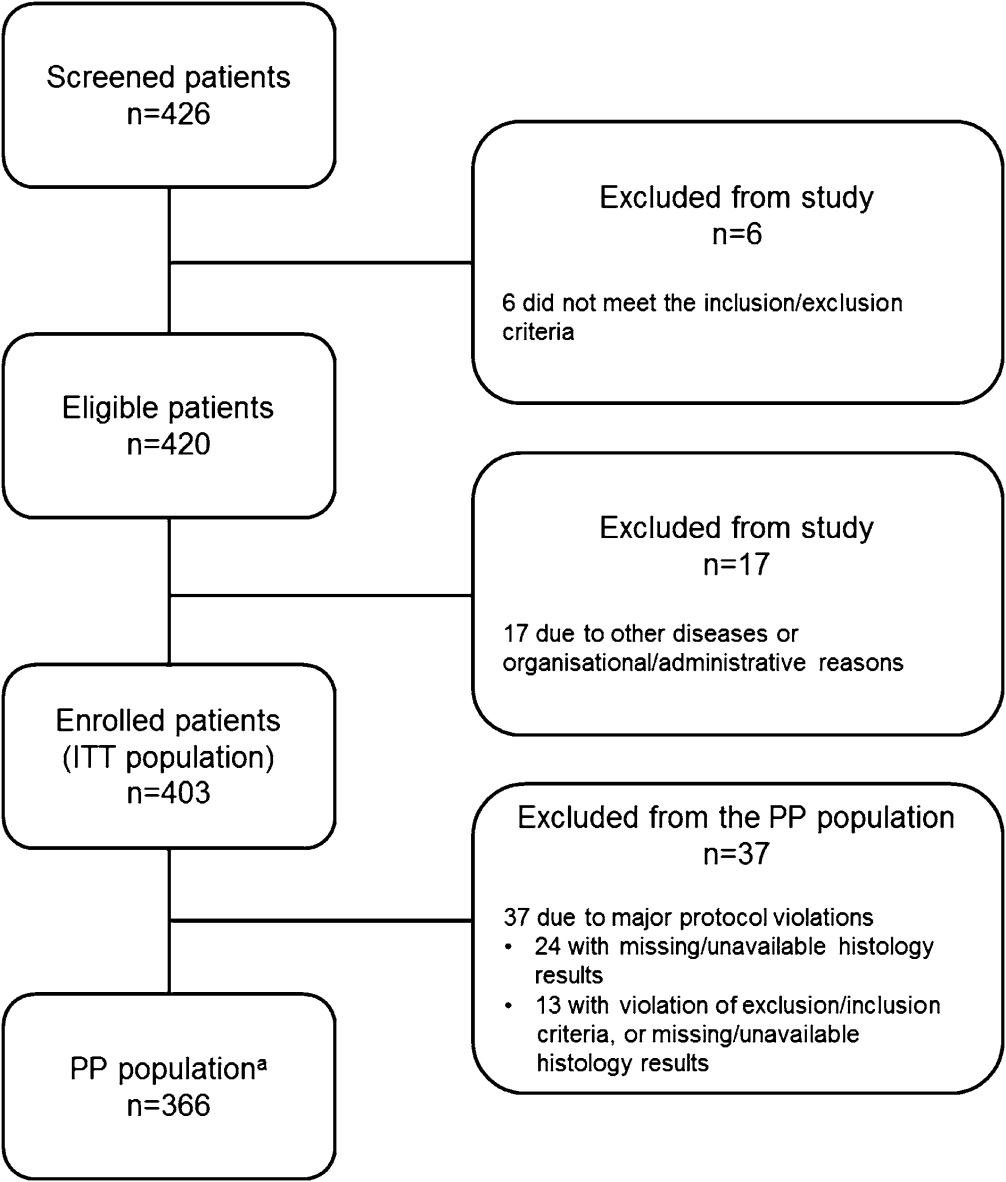
Participant flow chart. *ITT* intent to treat, *PP,* per protocol. ^a^Patients with available and completed histological data; 8 patients included in the PP population received WLC, but did not receive HALC, due to either technical problems or organisational issues

Participant flow is summarised in Fig. 1. Overall, 395 patients received hexaminolevulinate and 379 had valid histology from the diagnostic procedure; 97 % of enrolled patients completed the procedure.

Baseline patient and disease characteristics for the ITT population are summarised in Table 1.

**Table 1:** Baseline patient and disease characteristics (ITT population)

Characteristics	ITT population (*N* = 403)
Age (years)	
Mean (SD)	69.9 (10.7)
Sex, *n* (%)	
Female	114 (28.3)
Male	289 (71.7)
Current diagnosis is based on *n* (%)	
Positive urine cytology	34 (8.4)
Cystoscopy results	356 (88.3)
Other	65 (16.1)
Suspicion of NMIBC, *n* (%)	
Primary tumour	275 (68.2)
Recurrence	126 (31.3)
Missing	2 (0.5)
Suspicion of CIS, *n* (%)	
Yes	51 (12.7)
No	350 (86.8)
Missing	2 (0.5)
Suspicion of high-grade tumour, *n* (%)	
Yes	83 (20.6)
No	318 (78.9)
Missing	2 (0.5)
Previous BCG instillation, *n* (%)	20 (5.0)
Mean time between first diagnosis and end of the last BCG instillation (months)	27.1
EORTC recurrence risk^a,b^	
High risk	5 (2.3)
Intermediate risk	181 (83.4)
Low risk	31 (14.3)
Missing	65
EORTC progression risk^a,b^	
High risk	65 (30.0)
Intermediate risk	102 (47.0)
Low risk	50 (23.0)
Missing	65

*BCG* Bacillus Calmette–Guérin, *CIS* carcinoma in situ, *EORTC* European organisation for research and Treatment of cancer, *NMIBC* non-muscle invasive bladder cancer, *SD* standard deviation

^a^Percentages are calculated based on the number of histologically evaluated patients with cancerous lesions (*n* = 282) minus missing patients (*n* = 65), i.e. *N* = 217

^b^EORTC is only applicable to patients with cancerous lesions, with respective stage Ta, T1, and CIS

### Histology

Of the 941 biopsy samples collected, 929 were histologically evaluated. Of these, 876 (94.3 %; 95 % CI 92.6–95.7) were from lesions suspicious under WLC and/or HALC, while 53 (5.7 %; 95 % CI 4.3–7.4) were random samples taken from unsuspicious tissue and excluded from analyses. Results from histological analysis are summarised in Table 2.

**Table 2:** Summary of histology results (PP population)

Parameter statistic/value	*N* (%)
Biopsy samples collected (ITT population), *N*	941
Histologically evaluated samples (ITT population), *N*	929
Samples obtained from suspicious lesions, *n*	839
Histological findings^a^	
Carcinoma	499 (59.5)
Dysplasia	26 (3.1)
Hyperplasia	29 (3.5)
Healthy tissue	83 (9.9)
Inflammation	202 (24.1)
Unknown^b^	0 (0.0)
Missing	0 (0.0)
Tumour staging^c^	
Ta	310 (62.1)
T1	99 (19.8)
T2	48 (9.6)
T3	0 (0.0)
T4	0 (0.0)
CIS	36 (7.2)
Unknown	6 (1.2)
Grading WHO 1973^c^	
G1	130 (26.1)
G2	205 (41.1)
G3	108 (21.6)
Unknown	23 (4.6)
Not selected	33 (6.6)
Grading WHO 2004^c^	
PUNLMP	5 (1.0)
Low grade	251 (50.3)
High grade	146 (29.3)
Unknown	62 (12.4)
Not selected	35 (7.0)

*CIS* carcinoma in situ, *PUNLMP* papillary urothelial neoplasm of low malignant potential, *WHO* world health organisation

^a^Percentages are calculated based on the number of samples obtained from suspicious lesions (*n* = 839)

^b^Histology result is either “not selected” or “unknown”

^c^Only applicable to carcinoma lesions. Percentages are calculated based on the number of lesions found to be carcinoma (*n* = 499)

### Primary and secondary outcome measures

Of the 403 enrolled patients, 24 (6.0 %) were not submitted to cystoscopy or were without valid cystoscopy results (Fig. 1).

**Fig. 2:**
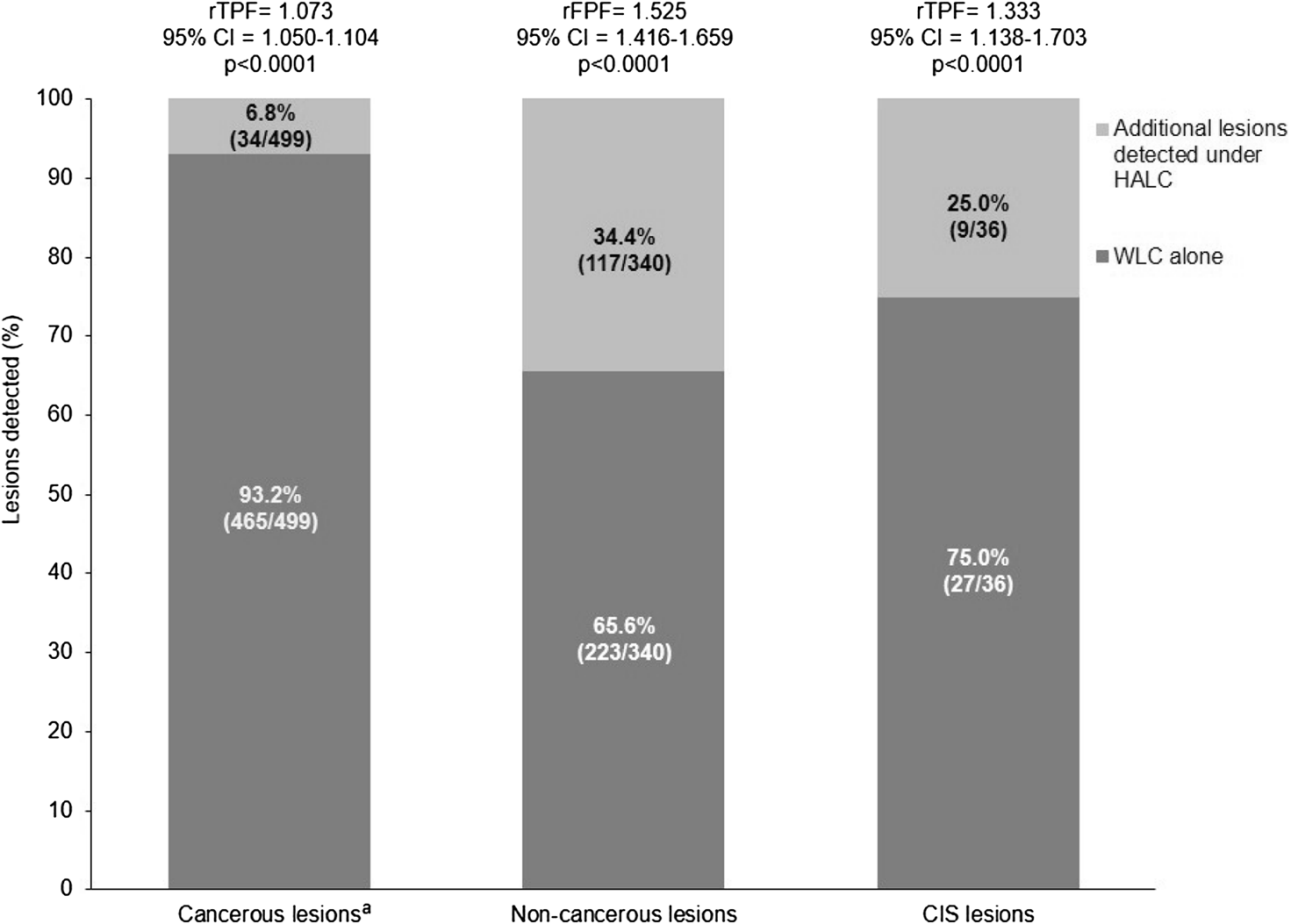
Histologically confirmed lesions detected with HALC and WLC (PP Population). *CIS* carcinoma in situ, *HALC* hexaminolevulinate blue light cystoscopy, *rFPF* ratio of false-positive fractions, *rTPF* ratio of true-positive fractions, *WLC* white light cystoscopy. By definition, the detection rate was 100 % for HALC plus WLC. ^a^2.8 % of cancerous lesions were detected by WLC only

Overall, 499 cancerous lesions and 340 non-cancerous lesions in the PP population were identified with HALC and WLC. HALC identified 6.8 % (34/499) more positive lesions than WLC (Fig. 2). The rTPF and rFPF indicated that HALC plus WLC detected statistically significantly more true-positive and false-positive lesions. From the 839 suspected lesions, 223 (26.6 %) were false positive under WLC and an additional 117 (13.9 %) were false positive under HALC.

Within the PP population, 270 (73.8 %) and 96 (26.2 %) NMIBC patients had histologically confirmed cancerous lesions and non-cancerous lesions, respectively. Of these patients, 234 (86.7 %; 95 % CI 82.0–90.5) had all confirmed tumour lesions detected with HALC and WLC, and 27 (10.0 %; 95 % CI 6.7–14.2) had ≥1 additional positive lesion detected with HALC. In the PP population, 2.2 % (95 % CI 0.8–4.8) of NMIBC patients would have been missed with WLC alone. In these patients, lesions with the following tumour stages were documented: two CIS, two pTaG1, four pTaG2, and two pT1G3.

On a patient level, and taking into account a study-design-related 100 % detection rate for HALC and WLC, detection rate was 95.4 % (95 % CI 92.7–97.3) for WLC alone. The resulting ratio of detection rates of 1.049 (95 % CI 1.028–1.079; *p* = 0.0027) indicated that a statistically significant increase in the number of patients with cancerous lesions was observed with WLC plus HALC versus WLC alone. The rFPF was 1.157 (95 % CI 1.080–1.283; *p* < 0.0001), indicating that with WLC plus HALC versus WLC alone a statistically significantly increased number of patients with false-positive lesions was observed.

With WLC and HALC, 12 (8.2 %) additional cancerous lesions were detected in patients at high risk of progression (according to EORTC scores) versus with WLC (*p* < 0.0001). No statistical difference was detected between HALC and WLC in those at low (*n* = 1 [2.1 %]; *p* = 1.0000) and intermediate (*n* = 8 [4.3 %]; *p* = 0.0711) risk of progression.

In a post hoc analysis, WLC plus HALC identified 9 (25.0 %) additional CIS lesions in the PP population versus WLC alone (Fig. 2), with the rTPF indicating that HALC detected statistically significantly more CIS lesions (*p* < 0.0001).

Based on the 282 patients in the ITT population with histologically confirmed lesions, post hoc analysis showed that 16 patients must be examined using HALC versus WLC alone to detect one additional patient with a cancerous lesion (irrespective of risk of recurrence or progression).

No AEs related to treatment with HALC were reported.

## Discussion

This study showed that in patients with NMIBC undergoing TURB in routine clinical practice, WLC plus HALC detected an additional 34 (6.8 %) cancerous lesions in the PP population versus WLC alone (*p* < 0.0001). While this proportion is markedly lower than in a previous observational study (23.2 %) [7], findings from both are consistent with results from RCTs (range 7–30 %) [6–11]. Large ranges in reported results could be due to different documentation procedures and clinical settings across countries. Nevertheless, HALC provides a diagnostic benefit over WLC alone for patients with suspected NMIBC, supporting its use in daily clinical practice. Indeed, the EAU guidelines now recommend use of this imaging modality when available for high-risk patients (when cytology is positive or when high-risk exophytic tumour is expected) [17]. The validity of this approach is confirmed by the current study, in which 8.2 % of additional cancerous lesions were detected in patients at high risk of progression. Initially targeting HALC to high-risk patients seems an appropriate means of introducing the use of this technique within a treatment centre. Thus, while HALC may lead to long-term benefits across all risk groups compared with WLC, high-risk patients seem to benefit most [16].

Importantly, significantly more CIS lesions were detected with WLC plus HALC versus WLC alone. While the effect of HALC was weaker than in a previous observational study, the rate of CIS was also lower (7.2 % here vs. 19.4 % in an observational series) [7]. RCTs also reported a greater (31.9–70.6 %) detection of additional CIS lesions with HALC [3].

In the PP population, 10 % of patients had ≥1 additional positive lesion detected with HALC, and WLC would have missed 2.2 % of NMIBC patients. Therefore, with WLC plus HALC versus WLC alone, more appropriate risk classification and optimised treatment management may be possible.

Comparisons with findings from RCTs should be viewed with caution. In RCTs, carefully selected patient populations managed at specialist centres with potentially more experienced surgeons may produce results that are not easily transferrable to routine practice at larger or more diverse centres. Indeed, TURB is a teaching procedure often performed by less experienced surgeons. Likewise, local pathology laboratories may yield different results, and generally a central pathology laboratory is used in RCTs. Hence, results from studies of routine HALC use are important to better understand the potential benefits of this approach. As outlined above, there is good evidence that HALC may offer benefits in terms of detection and outcome in a range of patients; this benefit may be most significant in those with high-risk tumours (as the data from this study support), and optimising detection of positive lesions will rely on optimising the technique more than via patient selection. Furthermore, differences in local histology/pathology assessments may contribute to varied results between observational studies and clinical trials; conformity in staging and grading is 50–60 % [1]. Importantly, this study included sites experienced and inexperienced with HALC, reflecting routine practice in Germany.

Enhanced detection during TURB with the use of HALC may improve diagnosis of NMIBC in clinical practice, improve outcomes, and reduce recurrence rates [4]. The finding that HALC detected a significantly higher rate of true-positive lesions in patients with high risk of progression (according to EORTC scores) than WLC may indicate that HALC use could improve detection of these patients in routine clinical practice, potentially reducing morbidity and mortality [8, 11, 17, 18]. While false-positive rates were relatively high with WLC alone and when HALC was added, these were within the ranges reported in a previous observational study that highlighted the wide variation in the rate of false positives at different centres (2.6–28.6 % with WLC alone and 6.1–39.3 % with the addition of HALC) [8]. The present study highlights the importance of thoroughly assessing patients in true need of a TURB in advance to avoid biopsy in merely inflamed tissue, as a rather large fraction of patients showed inflammation only (24.1 %).

Although this study did not assess recurrence rate or survival, it is clear that improved visualisation techniques allow for more appropriate disease staging and risk classification and thus guide more appropriate subsequent treatment decisions, as well as allow for better resection of the tumour. It is reasonable, therefore, to expect that use of HALC would result in longer recurrence-free survival compared to WLC alone. The benefits of improved detection with HALC have already been demonstrated in several studies, with significantly reduced recurrence rates at 1 year. This could potentially reduce the requirement for patients to undergo frequent TURBs, which not only have a significant negative impact on patient quality of life, but also incur a considerable cost [3]. A recent study from Sweden modelling the cost consequences of HALC found that the technique had a minimal cost impact if introduced across all risk groups, and reduced TURBs, cystectomies, bed days, and operating room time. Notably, the use of HALC translated into cost savings from year 2–5 in this model [16].

Despite the large number of patients with primary tumours (68.6 %) versus previous studies (43.3 %) [3], this study represents a typical population of patients with suspected diagnosis of NMIBC within in-patient surgical urological institutions in Germany. Similar to other observational studies, there were no reports of spontaneous AEs related to hexaminolevulinate [7, 8].

No subgroup analyses by tumour type were conducted in the present study; however, a meta-analysis that did conduct such analyses found the benefit of HALC was in patients with Ta, T1, CIS, primary and recurrent cancer, and was independent of the level of risk [3]. Additional detection rates with HALC (compared with WLC alone) ranged from 9.7 to 40.2 % for Ta tumours, with 239 of 1621 (14.7 %) of additional Ta tumours only being detected with HALC. For T1 tumours, detection of additional tumours with HALC ranged from 3.6 to 54.5 % of the total T1 tumours detected. In total, 40 of 372 (10.8 %) of additional T1 tumours were detected only by HALC. As in the present study, the benefit of HALC was most pronounced in CIS; detection of additional CIS lesions ranged from 31.9 to 70.6 % of the total CIS lesions detected; and 215 of 527 (40.8 %) of additional CIS lesions were detected with HALC alone [3].

Despite meticulous online documentation with detailed bladder maps and minimised time delay between procedures and documentation, there remained a number of study limitations. Limitations were consistent with other observational studies and include: incomplete information versus RCTs, and lack of blinding between WLC and HALC, which could bias true-positive and false-positive observations. However, achieving blinding in routine clinical settings is impossible and also hard to accomplish in a diagnostic study. Of note, no tertiary or academic centres—both of which institutions treat a significant number of patients—were included in the present study, and not all regions of Germany were equally reflected; either or both of these factors may have introduced bias.

Study strengths included observation of a complete and typical spectrum of patients with a realistic high number of primary tumours and staging distribution, and prevention of patient selection bias through recruitment of consecutive patients with the required diagnosis.

In conclusion, this study demonstrates that HALC significantly improves the detection of NMIBC versus WLC alone in daily clinical practice in Germany.
